# Mitochondrion-Mediated Cell Death through Erk1-Alox5 Independent of Caspase-9 Signaling

**DOI:** 10.3390/cells11193053

**Published:** 2022-09-29

**Authors:** Min Chen, Lei Wang, Min Li, Marietta M. Budai, Jin Wang

**Affiliations:** 1 Department of Pathology and Immunology, Baylor College of Medicine, Houston, TX 77030, USA; 2 Immunobiology and Transplant Science Center, Houston Methodist Research Institute, Houston, TX 77030, USA; 3 Department of Surgery, Weill Cornell Medical College, Cornell University, New York, NY 10065, USA

**Keywords:** caspase-9-independent cell death, Erk1, Alox5, ROS, lipid peroxidation

## Abstract

Mitochondrial disruption leads to the release of cytochrome c to activate caspase-9 and the downstream caspase cascade for the execution of apoptosis. However, cell death can proceed efficiently in the absence of caspase-9 following mitochondrial disruption, suggesting the existence of caspase-9-independent cell death mechanisms. Through a genome-wide siRNA library screening, we identified a network of genes that mediate caspase-9-independent cell death, through ROS production and Alox5-dependent membrane lipid peroxidation. Erk1-dependent phosphorylation of Alox5 is critical for targeting Alox5 to the nuclear membrane to mediate lipid peroxidation, resulting in nuclear translocation of cytolytic molecules to induce DNA damage and cell death. Consistently, double knockouts of caspase-9 and Alox5 in mice, but not deletion of either gene alone, led to significant T cell expansion with inhibited cell death, indicating that caspase-9- and Alox5-dependent pathways function in parallel to regulate T cell death in vivo. This unbiased whole-genome screening reveals an Erk1-Alox5-mediated pathway that promotes membrane lipid peroxidation and nuclear translocation of cytolytic molecules, leading to the execution of cell death in parallel to the caspase-9 signaling cascade.

## 1. Introduction

During intrinsic apoptosis, mitochondrial outer membrane permeabilization leads to the release of cytochrome c into the cytosol to form apoptosome with Apaf-1 and caspase-9, resulting in the activation of caspase-9 [1,2,3,4]. Caspase-9 is the initiator caspase that activates the downstream effector caspases, including caspase-3, caspase-6 and caspase-7 [5]. The cleavage of DFF45/ICAD by caspase-3 leads to the activation of DFF40/CAD that enters the nucleus to mediate DNA cleavage and apoptosis [6,7,8,9]. In addition to cytochrome c, Smac/DIABLO released from mitochondria can facilitate the activation of caspase-9 and caspase-3 by sequestering XIAP [10,11]. Moreover, AIF and endonuclease G (EndoG) have been shown to translocate from disrupted mitochondria to the nucleus to induce DNA damage [12,13]. In addition to caspase-dependent apoptosis, different non-apoptotic forms of cell death have been described [14]. In the absence of caspase activation, cell death signaling can induce Ripk1 and Ripk3 activation by autophosphorylation [15,16], followed by phosphorylation and activation of downstream pore-forming MLKL to trigger necroptosis [17,18]. Inflammasome-mediated activation of caspase-1 can induce the cleavage of gasdermin D (GSDMD), resulting in the generation of N-terminal pore-forming GSDMD-N to trigger pyroptosis [19]. Elevated intracellular iron and depletion of antioxidant glutathione can cause lipid peroxidation and ferroptosis, which is inhibited by glutathione peroxidase 4 (GPX4) [20,21,22].

Although the caspase-9-dependent pathway can rapidly trigger apoptosis after mitochondrial disruption, it appears that interruption of caspase-9-dependent apoptosis may shift cells to engage other cell death mechanisms. Caspase-9-knockout mice display perinatal lethality with prominent defects in brain development that may be associated with defective cell death in neuronal progenitor cells [23,24]. However, cell death in lymphocytes and other cell types in the absence of caspase-9 appears to be largely intact [25]. In addition, apoptosis is efficiently inhibited by the over-expression of Bcl-2, but not by the deletion of caspase-9 [26], suggesting that caspase-9-independent cell death mechanisms can efficiently carry out intrinsic apoptosis. Cells can often undergo caspase-independent cell death when caspase function is inhibited [27,28]. Erk-dependent induction of caspase-independent cell death has been shown in neuronal cells [29]. Induction of caspase-independent cancer cell death may also be a common feature for many chemotherapeutic drugs [30,31,32,33,34,35,36,37,38]. These studies indicate that caspase-9-independent mechanisms are involved in cell death under different physiological and pathological conditions.

Caspase-8-independent cell death mechanisms have been intensively studied. Rip1 and Rip3 have been shown to mediate necroptosis when caspase-8 activation is inhibited [15,16,17,39,40,41]. While the release of cytochrome c into the cytosol triggers caspase-9 activation [1,2,3,4], ROS production by mitochondria has been suggested to induce caspase-independent cell death [42]. However, genes that are critical for ROS generation to induce cell death independent of caspase-9 have not been defined. Moreover, downstream signaling events that mediate caspase-9-independent cell death have not been systemically characterized. Mitochondrion-dependent cell death is important for the maintenance of T cell homeostasis and immune tolerance [43]. Whether caspase-9 signaling downstream of the mitochondrion can regulate T cell functions and homeostasis has not been extensively studied due to perinatal lethality of caspase-9^−/−^ mice [23,24]. We therefore generated mice with conditional knockout of caspase-9 in T cells (T/casp9^−/−^). However, caspase-9^−/−^ T cells efficiently underwent cell death induced by different stimuli. Moreover, we did not detect T cell accumulation in T/casp9^−/−^ mice, suggesting that the existence of other cell death mechanisms independent of caspase-9 in T cells. To define the caspase-9-independent cell death pathway downstream of mitochondrion disruption, we performed a genome-wide siRNA library screening for genes involved in cell death using caspase-9-deficent Jurkat T cells. Our data suggest that mitochondrial disruption leads to Erk1-dependent phosphorylation and nuclear membrane translocation of Alox5, resulting in membrane lipid peroxidation, nuclear entry of nucleases and cytolytic molecules to induce cell death.

## 2. Materials and Methods

### 2.1. Mice

Lck-cre mice (The Jackson Laboratory) were bred with caspase-9^flox^ mice [28] to obtain Lck-cre/caspase-9^flox/flox^ mice with T cell-specific knockout of caspase-9 (T/casp9^−/−^). Alox5^−/−^ mice (The Jackson Laboratory) were crossed with T/casp9^−/−^ mice to obtain T/casp9^−/−^Alox5^−/−^ mice. Experiments were performed according to federal and institutional guidelines and with the approval of the Institutional Animal Care and Use Committee of Baylor College of Medicine (AN-2099) and the Houston Methodist Research Institute (IS00006582).

### 2.2. siRNA Screening

JMR cells [44], a caspase-9-deficient human T cell leukemia Jurkat cell line, cultured in 384-well plates were robotically transfected with 40 nM of a pool of 4 siRNAs targeting each of a total of 21,121 genes in the human genome (Dharmacon, Lafayette, CO, USA) by reverse transfection using RNAiMax (Life Technologies, Carlsbad, CA, USA) at the MD Anderson Cancer Center siRNA Screening Facility. siRNAs for each gene were transfected into JMR cells in 6 replicates of 384-well plates. In addition to the targeting siRNAs, each plate also contained transfection with non-targeting siRNA as negative control, as well as siRNA targeting PLK (siPLK) to induce cell death [45]. Forty-eight hours after transfection, the cells were cultured with 6 μM ABT-263 or solvent control in triplicates for another 48 h. Live cells were quantitated by the Celltiter-Fluor cell viability assay (Promega, Madison, WI, USA). The percentage of cell death induced by ABT-263 treatment was calculated as follows: (control-treated)/control × 100%. We scored a gene as a positive hit if the killing rate after its silencing is more than 2× standard deviation (SD) above or below the average killing rate on its plate. According to this standard, we identified 776 such genes in the first round of siRNA screening. In the second round of screening, four siRNAs targeting these 776 gens were transfected into JMR cells separately. Killing with ABT-263 and quantitation of cell viability was performed in the same way as in the first round of screening. The candidate genes identified in the second round of screening were used for further analyses. The candidates for cell death and anti-cell death genes are listed in Tables S1 and S2. To characterize the function of some of these candidate genes identified in the second round of screening, two to three validated siRNAs targeting selected genes were obtained from Life Technologies and used for transfection of JMR cells at a final concentration of 100 nM and used for various analyses. The sequences for these siRNAs are listed in Table S3.

### 2.3. Cell Death Assay

Cells were cultured in 96-well tissue culture plates in the presence or absence of indicated treatments for different time. The cells were stained with APC-annexin V (ThermoFisher, Waltham, MA, USA) and 5 μg/mL propidium iodide (PI) (ThermoFisher), followed by flow cytometry analyses. Percentage of cell death induced by treatments with different apoptosis stimuli was calculated from live cells of untreated and treated groups as follows: (untreated-treated)/treated × 100%. In some experiments, 10 μM necrostatin-1, necroX-5 (Enzo Life Sciences, Farmingdale, NY, USA) or carbobenzoxy-valyl-alanyl-aspartyl-O-methyl-fluoromethylketone (zVAD, Promega) was included in the culture. Cells were also stained with APC-conjugated Annexin V (BD Bioscience, San Jose, CA, USA) in some assays. Mouse T cells were stimulated cultured with 5 μg/mL Concanavalin A (con A) ConA and 100 U/mL IL-2 for 48 h and used for different assays.

To determine the effects of phosphorylation of Alox5, substitution of Serine 271 to Alanine (S271A) in Alox5 was performed using the site-directed mutagenesis kit (Stratagene, San Diego, CA, USA). Plasmids expressing Alox5-GFP (Origene, Rockville, MD, USA), Alox5^S271A^-GFP or GFP alone were transfected into JMR cells by electroporation using the Neon transfection system (Life Technologies). After 24 h culture, the cells were treated with 6 μM ABT-263 or solvent control. The loss of live GFP^+^ cells after ABT-263 treatment was determined 24 h later.

### 2.4. Measurement of ROS and Lipid Peroxidation

To measure ROS, JMR cells were cultured in the presence or absence of ABT-263 for 12 h. The cells were then incubated with 5 μM Mito-SOX (Life Technologies) at 37 °C for 30 min. In some experiments, cells were also stained with APC-annexin V, followed by flow cytometry. To determine lipid peroxidation, JMR cells with or without ABT-263 treatment as above were incubated with 5 μM BODIPY 581/591 C11 (Life Technologies) at 37 °C for 1 h and analyzed by flow cytometry.

### 2.5. Immunocytochemistry

Cells treated with cell death stimuli for 12 h were added to glass slides by cytospin and incubated with monoclonal mouse anti-TIA-1 (Abcam, Boston, MA, USA), monoclonal mouse anti-AIF (Santa Cruz Biotechnologies, Dallas, TX, USA) or polyclonal rabbit anti-EndoG (ProSci, Fort Collins, CO, USA). The cells were then incubated with Alexa Fluor 488-conjugated goat anti-mouse or anti-rabbit IgG (Life Technologies). The nuclei were counterstained with DAPI (Thermo Scientific). Cells treated with cell death stimuli for 24 h were used for TUNEL staining using the Click-iT TUNEL assay kit (Life Technologies). The nuclei were counterstained with DAPI. Alox5-GFP or Alox5^S271A^-GFP expression plasmids were transfected into JMR cells by electroporation using the Neon transfection system (Life Technologies) and cultured for 24 h. The cells were then treated with ABT-263 or solvent control for 8 h, followed by cytospin onto glass slides and DAPI staining. The staining was examined using a Nikon Eclipse 80i fluorescence microscope.

### 2.6. Immunoprecipitation and Western Blot

pCMV6-Alox5-GFP (Origene) was transfected into 293T cells with vectors expressing FLAG-tagged MAPKs. The cells were lysed 24 h later and immunoprecipitated with anti-GFP (Origene), followed by Western blot with anti-phospho-Alox5^S217^ (Cell Signaling Technology, Danvers, MA, USA). The blots were also probed with anti-GFP. Total lysates were used for Western blot by probing with anti-FLAG (Sigma, St. Louis, MO, USA). JMR cells transfected with siRNA targeting specific genes or non-targeting siRNA control were lysed for Western blot by probing with specific antibodies. The following antibodies were used for Western blot: rabbit polyclonal or monoclonal antibodies to Alox5, ERK1, p38 MAPK, p44/42 MAPK (ERK1/2), phosphor-p44/p42 MAPK (p-ERK1/ERK2), RAD51, SAPK/JNK, SESN2 (Cell Signaling Technology), Alox5AP, DHODH, HADHA, MGST1, TIA-1 (Abcam), EndoG (ProSci), OXR1 (Bethyl Laboratory, Montgomery, TX, USA.) and PDP1 (Sigma); and mouse monoclonal antibodies to AIFM1, API5, PNKP (Santa Cruz Biotechnology), caspase-3, caspase-6 caspase-7, caspse-9, phosphor-p38 MAPK, phosphor-SPAK/JNK (Cell Signaling Technology), FLAG (Sigma) and GFP (Origene). The blots were also probed with mouse monoclonal anti-α tubulin (Santa Cruz Biotechnology) to ensure equal loading.

### 2.7. Statistic Analyses

Data are presented as mean ± SD, and *p* values were determined by a two-tailed Student’s *t*-test using the GraphPad Prism software.

## 3. Results

### 3.1. Caspase-9-Independent Mechanisms Efficiently Mediate Mitochondrion-Dependent Cell Death and Maintain T Cell Homeostasis

**Figure 1:**
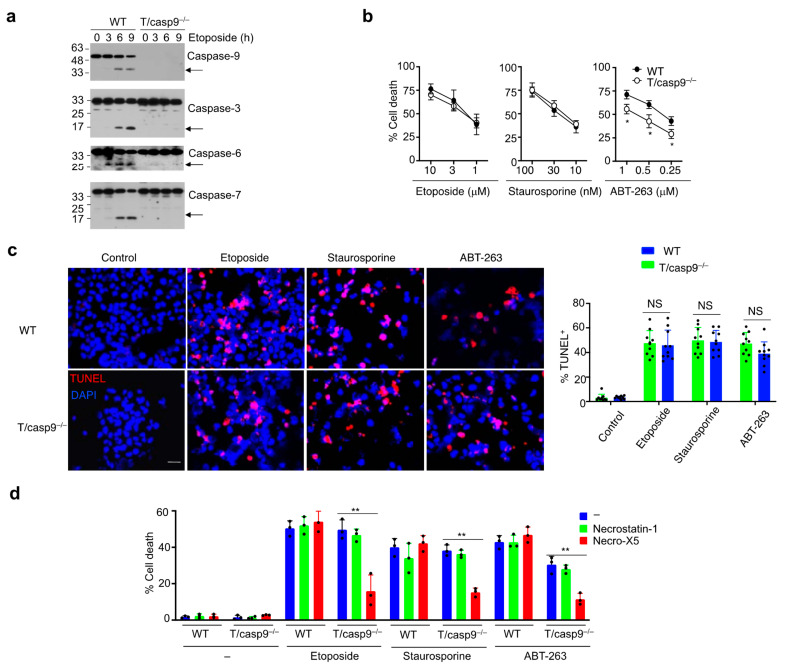
Cell death in caspase-9-deficient T cells. (**a**) T cells from wild-type (WT) or T/caspase-9^−/−^ (T/casp9^−/−^) mice were treated with etoposide and used for Western blot. Arrows indicate processed caspases. (**b**) Cell death in WT and caspase-9^−/−^ T cells triggered by different apoptosis stimuli for 24 h. Data are presented as mean ± SD. Comparison to control: * *p* < 0.05. (**c**) WT or caspase-9^−/−^ T cells treated with 1 μM etoposide, 30 nM staurosporine or 1 μM ABT-263 for 24 h were used for TUNEL staining (red) and nuclear counterstaining with DAPI (blue). Scale bar: 10 μm. Comparison to control: NS: Statistically not significant. (**d**) Cells were treated with cell death stimuli as in (**c**) with or without 10 μM necrostatin-1 or necroX-5. Percentages of cell death are presented as mean ± SD. Comparison to control: ** *p* < 0.01.

To define the roles for caspase-9-dependent intrinsic cell death in the regulation of T cell apoptosis and functions, we crossed Caspase-9^flox^ mice [28] with lck-cre mice to generate T cell-specific knockout of caspase-9 (T/casp9^−/−^). As expected, caspase-9 deletion led to virtually complete suppression of activation of effector caspases, including caspases-3, -6 and -7, in T cells treated with etoposide (Figure 1a). Despite the lack of caspase activation, cell death could be induced efficiently in T cells by etoposide (Figure 1b). Similarly, cell death could be induced efficiently in caspase-9-deficient T cells by another apoptosis inducer, staurosporine (Figure 1b). In treatments with etoposide, staurosporine also induced DNA fragmentation by TUNEL staining in both caspase-9^−/−^ T cells and wild-type controls (Figure 1c), indicating that cell death in the absence of caspase-9 also involves nuclear DNA damage. These results suggest that cell death can proceed efficiently in the absence of caspase-9 signaling cascade.

We also treated T cells with ABT-263, a BH3-mimetic that induces mitochondrion-dependent apoptosis by specifically inhibiting Bcl-2 and Bcl-xL [46]. Compared to wild-type controls, caspase-9^−/−^ T cells showed a small but reproducible decrease in killing by ABT-263 (Figure 1b). However, DNA fragmentation as shown by TUNEL staining was not significantly affected by caspase-9 deficiency (Figure 1c), suggesting that alternative mechanisms efficiently mediate DNA cleavage independent of caspase-9. This suggests that inhibition of Bcl-2 and Bcl-xL with ABT-263 triggers both caspase-9-dependent and -independent cell death. Why caspase-9^−/−^ T cells showed decreased killing by ABT-263, but not by etoposide or staurosporine is not entirely clear. It is possible that ABT-263 induces cell death exclusively through mitochondria and is more severely influenced by caspase-9 deficiency. However, knockout of caspase-9 did not cause T cell expansion in T/casp9^−/−^ mice (Figure S1), suggesting that caspase-9-independent cell death mechanisms are sufficient for the maintenance of T cell homeostasis in vivo.

We found that a cell death inhibitor targeting oxidative stress, necroX-5 [47], significantly inhibited cell death induced by etoposide, staurosporine or ABT-263 in caspase-9^−/−^ but not in wild-type T cells (Figure 1d). In contrast, a RIP1-specific necroptosis inhibitor, necrostatin-1 [48], did not inhibit cell death in caspase-9^−/−^ T cells induced by these apoptosis stimuli (Figure 1d). In addition, ABT-263-induced cell death was partially inhibited by the pan-caspase inhibitor zVAD in wild-type but not in caspase-9^−/−^ T cells (Figure S2a), indicating that cell death observed in caspase-9^−/−^ T cells is caspase independent. Interestingly, wild-type and caspase-9^−/−^ T cells that underwent ABT-263-induced cell death displayed positive staining by both Annexin V and Mito-SOX (Figure S2s). These data suggest that oxidative stress is required for cell death in caspase-9^−/−^ T cells but dispensable for that in wild-type T cells.

### 3.2. Genome-Wide siRNA Screening for Genes That Regulate Caspase-9-Independent Cell Death

**Figure 2:**
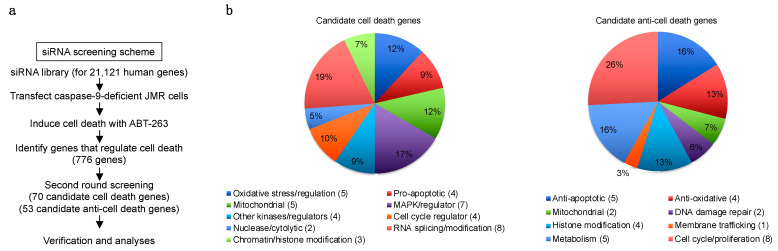
Genome-wide siRNA library screening for caspase-9-independent cell death genes. (**a**) A flow chart for the siRNA library screening of caspase-9-independent cell death genes. JMR cells were transfected with siRNA targeting 21,121 genes individually. (**b**) Pie charts for candidate cell death genes and anti-cell death genes identified after the second round of siRNA library screening. The complete lists of these genes are shown in Tables S1 and S2.

We next characterized caspase-9-independent cell death mechanisms downstream of mitochondrial disruption using a caspase-9-deficient human T cell leukemia Jurkat cell line, JMR [44]. JMR cells can be readily transfected with siRNA to silence target genes, Bax and Bak (Figure S3). Cell death in JMR cells induced by an inhibitor for Bcl-2 and Bcl-xL, ABT-263 [46], was suppressed by silencing of BAX and BAK (Figure S3). To identify genes that either inhibit or enhance ABT-263-induced cell death in caspase-9-deficient JMR cells, we used a siRNA library targeting of 21,121 genes of the human genome (Dharmacon) to perform a genome-wide screening (Figure 2a). The pools of four siRNA oligos targeting each of the genes were transfected into JMR cells in six replicates of 67 sets of 384-well plates. Forty-eight hours later, the transfected cells were treated with either ABT-263 or solvent control in triplicates for another 48 h, followed by measurement of viability. The percentage of killing of JMR cells induced by ABT-263 was calculated for cells transfected with siRNAs targeting each gene. We scored a gene as a positive hit if the killing rate after its silencing is more than 2× the standard deviation (SD) above or below the average killing on its plate. We identified 776 such candidate genes in the first screening. siRNA oligos targeting each of these genes were transfected separately into JMR cells to perform the second round of screening. A gene was scored as positive if at least two out of four of its siRNAs significantly increased or decreased ABT-263-induced killing of JMR cells. We identified 70 candidate pro-cell death genes whose silencing decreased ABT-263-induced killing of JMR cells (Figure 2b, Table S1). We also found 53 candidate anti-cell death genes whose silencing increased ABT-263-induced cell death (Figure 2b, Table S2). Interestingly, positive hits for candidate pro-cell death genes include oxidative stress-related genes, an endonuclease, MAP kinases and previously identified pro-apoptotic genes (Figure 2b, Table S1). Besides previously identified anti-apoptotic genes, positive hits for candidate anti-cell death genes include anti-oxidative genes and those genes encoding DNA damage repair enzymes (Table S2). These results support the potential roles for oxidative stress and DNA damage in mediating caspase-9-independent cell death.

### 3.3. Mitochondrial Proteins Essential for ROS Production during Caspase-9-Independent Cell Death

**Figure 3:**
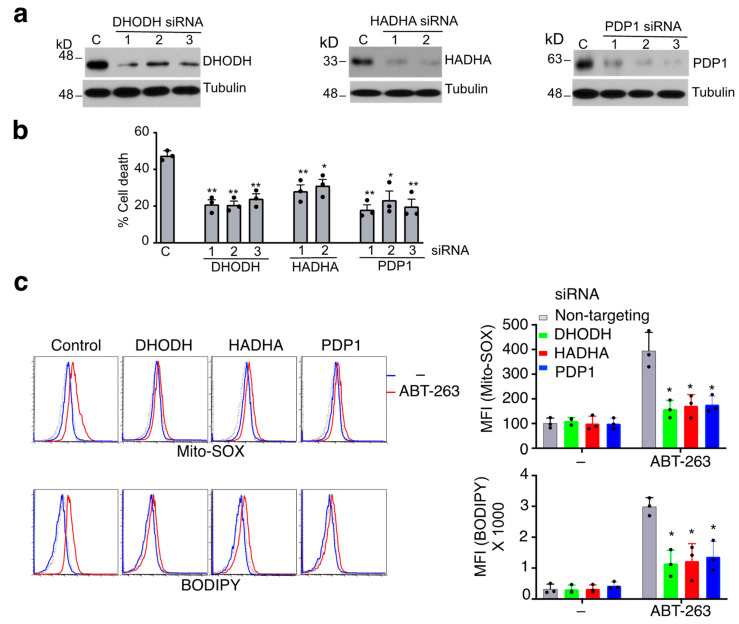
Genes involved in ROS and caspase-9-independent cell death. (**a**) JMR cells transfected with individual siRNA targeting different genes or non-targeting control (C), followed by Western blot analyses. (**b**) The cells were treated with ABT-263, followed by quantitation of cell death. (**c**) Cells transfected as in (**a**) were cultured with or without ABT-263. The cells were stained with Mito-SOX to measure ROS, or with BODIPY 481/581 C11 to measure lipid peroxidation, and analyzed by flow cytometry. Dashed line: unstained control. Mean fluorescence intensity (MFI) of the staining is presented as mean ± SD. * *p* < 0.05, ** *p* < 0.01.

In addition to pro-apoptotic BAX, other genes encoding mitochondrial proteins whose silencing suppressed cell death were also identified in the initial screening (Table S1). Transfection of individual validated siRNAs (Table S3) confirmed that dihydroorotate dehydrogenase (DHODH), 3-hydroxyacyl-CoA dehydrogenase Trifunctional Multienzyme Complex Subunit Alpha (HADHA) and pyruvate dehydrogenase phosphatase catalytic subunit 1 (PDP1), which are associated with respiratory chain, fatty acid oxidation or glucose metabolism [49,50,51], were involved in ABT-263-induced cell death in JMR cells (Figure 3a,b). This indicates that the mitochondrial proteins encoded by these genes promote caspase-9-independent cell death signaling after mitochondrial disruption.

Because caspase-9-independent cell death involves oxidative stress (Figure 1d), we determined whether these mitochondrial proteins are required for ROS generation during mitochondrion-dependent cell death. The mitochondrial pyrimidine biosynthesis enzyme DHODH, which is associated with mitochondrial electron transport, has been linked to ROS production and apoptosis [49,52], but the roles of other genes in cell death are unknown. Treatment with ABT-263 increased ROS production as measured by Mito-SOX staining in JMR cells transfected with a control siRNA (Figure 3c). Silencing DHODH, HADHA or PDP1 suppressed such increases in ROS (Figure 3c), suggesting that these genes are indeed involved in ROS production during caspase-9-independent cell death. Increased ROS production may cause membrane lipid peroxidation [53,54]. We therefore measured the levels of membrane lipid peroxidation in JMR cells by staining with BODIPY 581/591 C11 [55]. We found that treatment of JMR cells with ABT-263 indeed induced lipid peroxidation as measured by BODIPY staining, while silencing of DHODH, HADHA or PDP1 inhibited ABT-263-induced lipid peroxidation (Figure 3c). These data suggest that several mitochondrial proteins, including DHODH, HADHA and PDP1, are involved in promoting ROS production and membrane lipid peroxidation during caspase-9-independent cell death.

### 3.4. Protection against Caspase-9-Independent Cell Death by Genes That Inhibit Oxidative Stress

**Figure 4:**
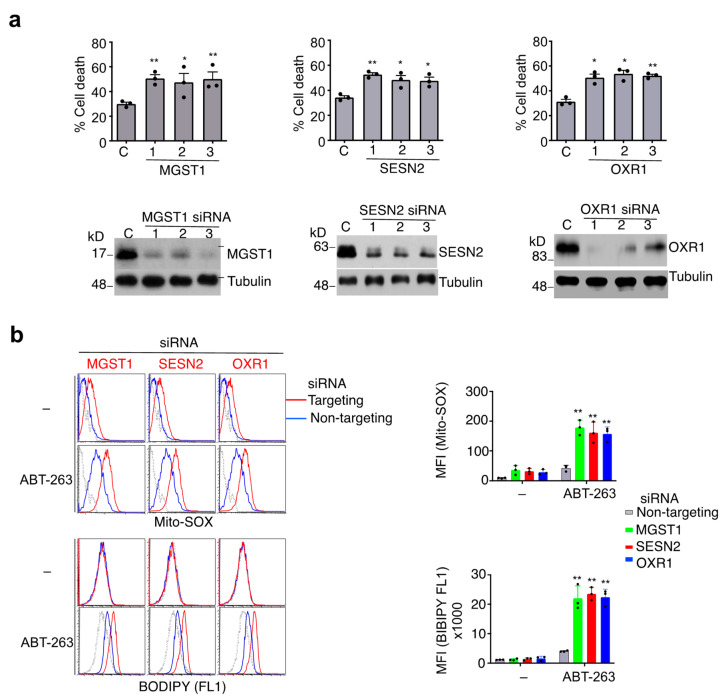
Genes that inhibit ROS and caspase-9-independent cell death. (**a**) JMR cells transfected with individual siRNA targeting different genes or non-targeting control (C). The cells were treated with ABT-263, followed by quantitation of cell death. The cells were also used for Western blot analyses. (**b**) Cells transfected as in (**a**) were cultured with or without ABT-263. The cells used for staining with Mito-SOX or BODIPY 481/581 C11. Dashed line: unstained control. Data are presented as mean ± SD. * *p* < 0.05, ** *p* < 0.01.

Because oxidative stress appears to be important for the induction of caspase-9-independent cell death, genes with pro-survival functions may function by protecting against oxidative stress. Indeed, some candidate anti-cell death genes identified in our screening have anti-oxidative stress functions (Table S2), including microsomal glutathione transferase 1 (MGST1), sestrin 2 (SESN2) and oxidative resistance 1 (OXR1). MGST1, SESN2 and OXR1 all have been implicated in the protection against oxidative stress and cell death by regulating glutathione metabolism or ROS scavenging [56,57,58,59,60]. We found that silencing of these genes increased the sensitivity of JMR cells to ABT-263-induced cell death (Figure 4a). Treatment with ABT-263 increased ROS levels in JMR cells by Mito-SOX staining, while silencing of these genes further promoted ROS production (Figure 4b, upper panels). Treatment with ABT-263 induced lipid peroxidation as measured by BODIPY staining, while silencing of MGST1, SESN2 or OXR1 further increased membrane lipid peroxidation (Figure 4b, lower panels). These results indicate that MGST1, SESN2 and OXR1 may inhibit caspase-9-independent cell death by inhibiting ROS production and lipid peroxidation.

### 3.5. Alox5-Mediated Lipid Peroxidation in the Induction of Caspase-9-Independent Cell Death

**Figure 5:**
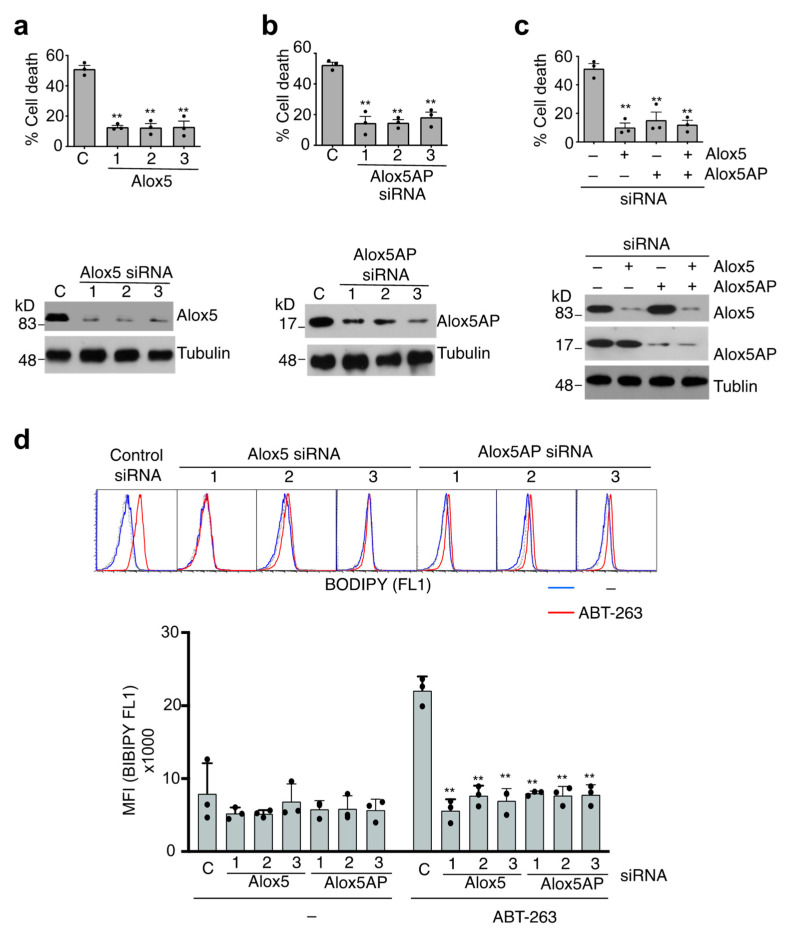
Alox5 and Alox5P in mediating caspase-9-independent cell death. (**a**–**c**) JMR cells transfected with non-targeting control siRNA (C) or individual siRNA targeting (**a**) Alox5 or (**b**) Alox5AP or (**c**) siRNA #1 targeting Alox5 and siRNA #1 targeting Alox5AP. The cells were treated with ABT-263, followed by quantitation of cell death. The cells were also used for Western blot analyses. (**d**) JMR cells transfected with siRNA targeting Alox5 or Alox5AP as in (**c**) were cultured with or without ABT-263, followed by staining with BODIPY 581/591 C11 and analyses by flow cytometry. Dashed line: unstained control. Data are presented as mean ± SD. Comparison with control siRNA group: ** *p* < 0.01.

Whether the induction of lipid peroxidation might be important for caspase-9-independent cell death is not known. Alox5, a lipoxygenase that promotes membrane lipid peroxidation and leukotriene biosynthesis [61,62], was identified as a potential caspase-9-independent cell death gene in our screening (Table S1). Interestingly, Alox5 has been implicated to play an important role in mediating cell death signaling in apoptosis, pyroptosis and ferroptosis [63]. We found that silencing of Alox5 significantly suppressed ABT-263-induced cell death (Figure 5a). Alox5-activating protein (Alox5AP), a nuclear membrane protein that promotes the enzymatic activity of Alox5 [64], was also a positive hit in our screening (Table S1). Silencing of Alox5AP also suppressed ABT-263-induced cell death (Figure 5b), while silencing of Alox5 and Alox5AP together did not further inhibit cell death (Figure 5c). This indicates that Alox5AP functions in the same pathway as Alox5 to mediate cell death. Silencing of Alox5 or Alox5AP blocked the induction of lipid peroxidation in JMR cells (Figure 5d), indicating that Alox5 and Alox5AP are required for mediating lipid peroxidation during caspase-9-independent cell death in JMR cells. These data suggest that Alox5-dependent lipid peroxidation contributes to caspase-9-independent cell death.

### 3.6. Phosphorylation of Alox5 Serine 271 by Erk1 Is Critical for Its Nuclear Membrane Localization and Cell Death Function

**Figure 6:**
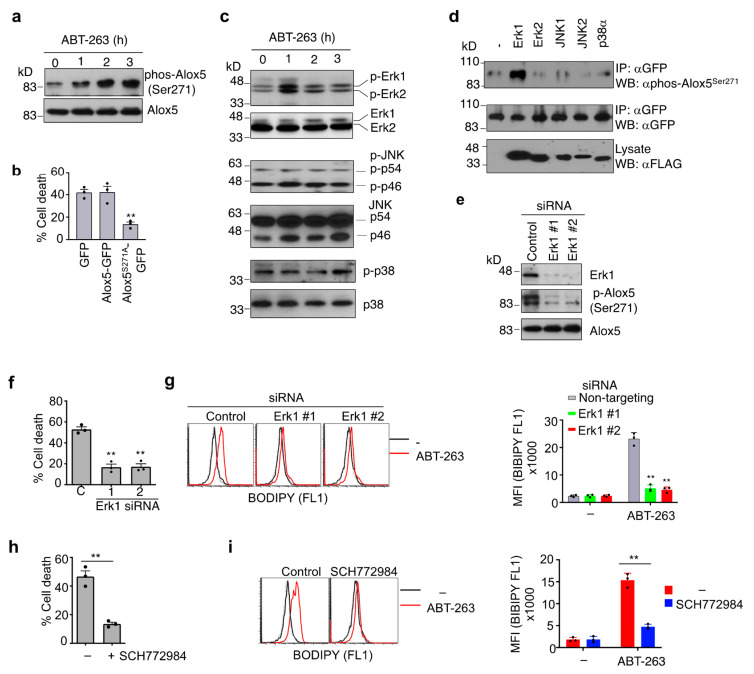
Regulation of Alox5 phosphorylation by Erk1. (**a**) JMR cells treated with ABT-263 for 0, 1, 2 and 3 h were lysed for Western blot analyses of endogenous Alox5 phosphorylation (p-Alox5) at Serine 271. (**b**) JMR cells transfected with plasmids expressing Alox5-GFP, Alox5^S271A^-GFP or GFP only were treated with ABT-263 for 24 h. The loss of live GFP^+^ cells was quantitated 24 h later. Data are presented as mean ± SD. Comparison to GFP control: ** *p* < 0.01. (**c**) JMR cells treated as in (A) were lysed for Western blot analyses of MAPKs. (**d**) Alox5-GFP was co-transfected with FLAG-tagged MAPKs into 293T cells. Cells were lysed 24 h later for immunoprecipitation with anti-GFP, followed by Western blot to detect phospho-Alox5^ser271^. (**e**) JMR cells transfected with siRNA targeting Erk1 were cultured in the presence of ABT-263 for 2 h, followed by Western blot to detect phosphorylation of Alox5 at Serine 271, or total Alox5 and ERK1. (**f**,**g**) Cells transfected with siRNA as in (**e**) were (**f**) cultured with ABT-263 for 24 h to analyze cell death or (**g**) stained with BODIPY 581/591 C11. Percentages of cell death and MFI of BODIPY 581/591 C11 staining are presented as mean ± SD. Comparison to the control siRNA group: ** *p* < 0.01. (**h**,**i**) JMR cells were treated with ABT-263 in the presence or absence of 5 μM SCH772984, followed by (**h**) analyses of cell death or (**i**) staining with BODIPY 581/591 C11. ** *p* < 0.01.

It has been shown that phosphorylation of Alox5 at Serine 271 affects nuclear membrane localization of Alox5 [65,66]. Whether phosphorylation regulates the cell death function of Alox5 is unknown. Interestingly, treatment of JMR cells with ABT-263 for 2 h led to increased phosphorylation of Serine 271 residue in Alox5 (Figure 6a). To investigate whether Alox5 phosphorylation might be important for Alox5 to induce cell death, we substituted Serine 271 of Alox5 with Alanine (Alox5^S271A^) in an Alox5-GFP fusion construct. Interestingly, transfection with Alox5^S271A^-GFP significantly suppressed ABT-263-induced cell death in JMR cells, whereas wild-type Alox5-GFP had no such effect (Figure 6b). This suggests that expressing more wild-type Alox5 is not sufficient to induce cell death, while modification by phosphorylation is required for its cell death activity. Overexpressed Alox5 mutant may compete with endogenous wild-type Alox5 for interaction with signaling molecules in this cell death pathway and inhibit the transmission of cell death signaling.

**Figure 7:**
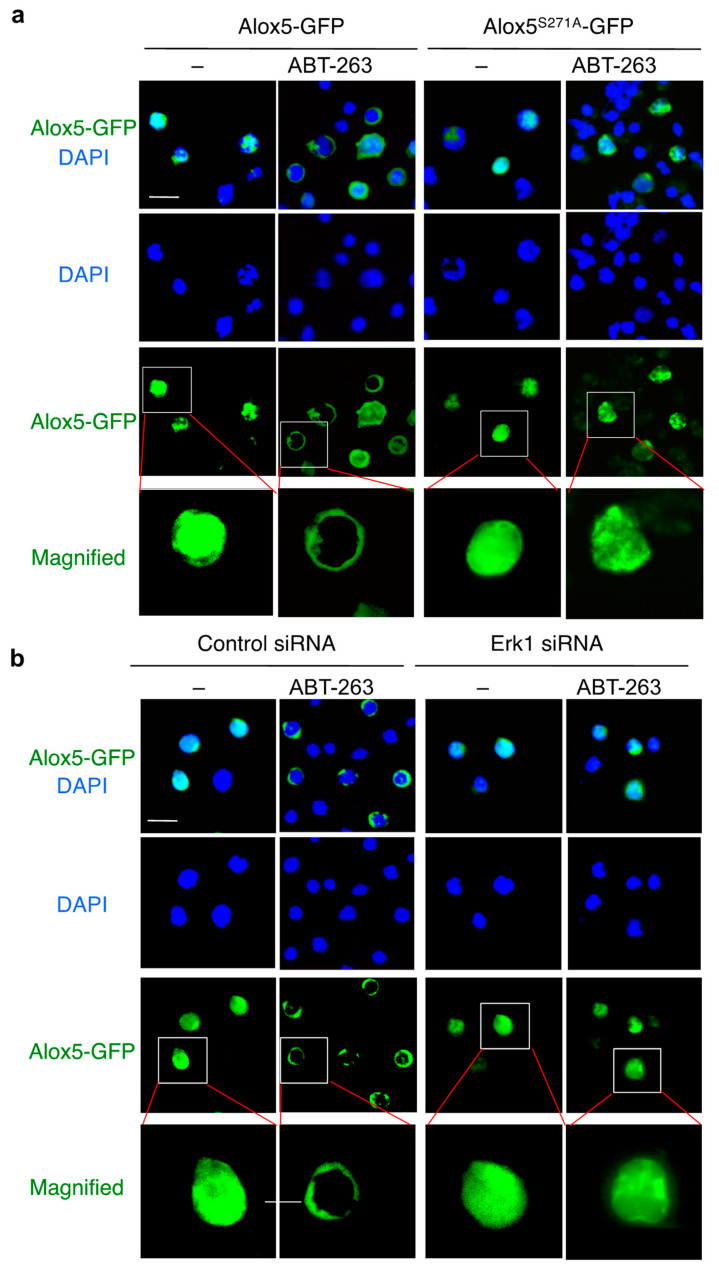
Regulation of Alox5 nuclear localization by Erk1. (**a**) JMR cells were transfected with Alox5-GFP or Alox5^S271A^-GFP. After 24 h culture, the cells were treated with ABT-263 for 9 h. The cells were added to slides by cytospin and stained with DAPI, followed by fluorescent microscopy. Scale bar, 10 μm. (**b**) JMR cells were transfected with Erk1 or control siRNA. After 24 h culture, the cells were transfected with Alox5-GFP and cultured for another 24 h. The cells were then treated with ABT-263 for 9 h and analyzed by fluorescent microscopy. Scale bar, 10 μm.

MAP kinases play an important role in the regulation of cell survival and cell death [67,68]. Our siRNA library screening identified several MAPK network members as candidate genes involved in caspase-9-independent cell death, including Erk1/MAPK3, MEKK3/MAP3K3 and ERK3/MAPK6 (Table S1). We therefore investigated whether MAPKs might be involved in Serine phosphorylation of Alox5. We first determined which MAPKs might be activated after the induction of caspase-9-independent cell death by measuring their phosphorylation. Among three major mammalian MAPK subfamilies: extracellular signal-regulated kinase (Erk), c-Jun NH2 terminal kinase (JNK) and p38 kinase, we detected activation of Erk1/2, but not JNK1, JNK2 or p38, as early as one hour after treatment with ABT-263 (Figure 6c). The activation of Erk1/2 preceded Alox5-Ser271 phosphorylation (Figure 6a,c), we therefore examined whether Erk1/2 could phosphorylate Alox5. We co-transfected Alox5-GFP with Erk1, Erk2, JNK1, JNK2 and p38. We found that co-transfection with Erk1, but not Erk2 or the other MAPKs, significantly increased Alox5 phosphorylation at Ser271 (Figure 6d). Silencing of Erk1 inhibited Alox5-Ser271 phosphorylation (Figure 6e). Moreover, silencing of Erk1 inhibited ABT-263-induced cell death (Figure 6f) and lipid peroxidation in JMR cells (Figure 6g). Consistently, an Erk1/2 inhibitor, SCH772984 [69], also potently inhibited the induction of cell death (Figure 6h) and lipid peroxidation (Figure 6i) in JMR cells treated with ABT-263. These results suggest that Erk1 is activated after mitochondrial disruption, leading to phosphorylation of Alox5 at Serine 271 and the induction of cell death. Alox5 has been shown to migrate from the nucleus or cytoplasm to the nuclear membrane after activation [70]. However, whether such translocation to the nuclear membrane takes place during caspase-9-independent cell death is not known. We observed that Alox5-GFP was localized to the nucleus in JMR cells (Figure 7a). Interestingly, Alox5-GFP showed perinuclear localization after treatment with ABT-263 (Figure 7a), suggesting that Alox5 is translocated to the nuclear membrane during caspase-9-independent cell death. In contrast, Alox5^S271A^-GFP did not show nuclear membrane translocation after treatment with ABT-263 (Figure 7a), indicating Ser271 phosphorylation is important for such translocation during the induction of cell death. We also found that silencing of Erk1 inhibited perinuclear translocation of Alox5-GFP after ABT-263 treatment (Figure 7b). Together, these data suggest that Erk1 is required for phosphorylation of Alox5 at Ser271, leading to nuclear membrane translocation of Alox5 and the induction of membrane lipid peroxidation.

### 3.7. Loss of Alox5 Inhibits Membrane Lipid Peroxidation and Cell Death in Caspase-9^−/−^ T Cells

**Figure 8:**
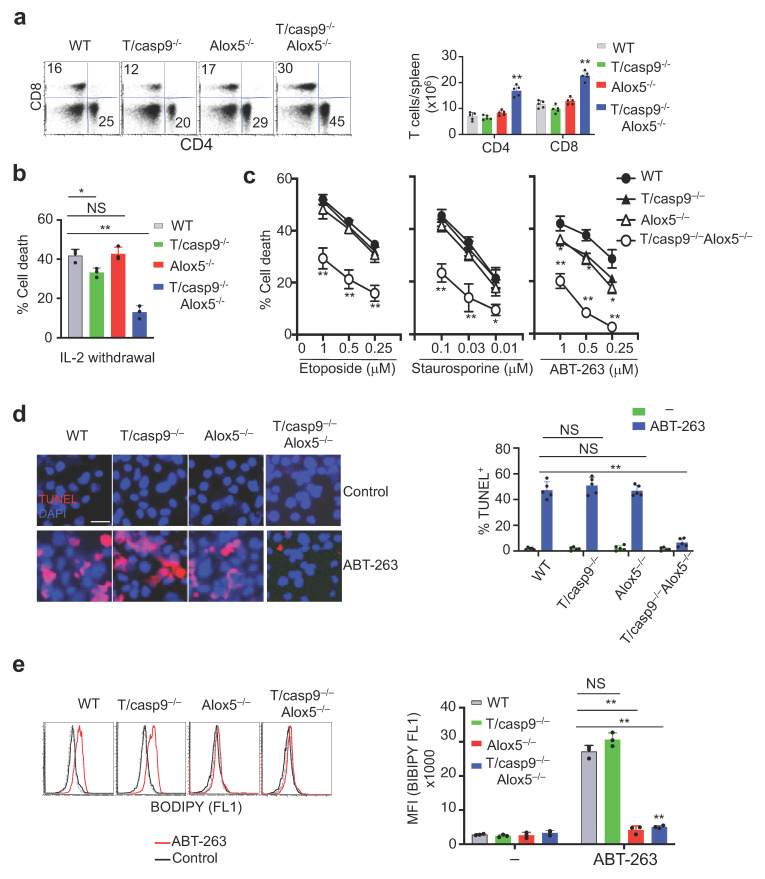
Inhibition of cell death by double knockouts of caspase-9 and Alox5 in T cells. (**a**) Flow cytometry analyses of CD4^+^ and CD8^+^ T cells in the spleen of 2-month-old T/caspase-9^−/−^Alox5^−/−^, T/caspase-9^−/−^, Alox5^−/−^ or wild-type (WT) mice (upper panel). (**b**,**c**) T cells activated with ConA and IL-2 were cultured in (**b**) the absence of IL-2, or (**c**) treated with indicated apoptosis stimuli for 24 h. Percentages of cell death are presented as mean ± SD. Comparison to wild-type control: * *p* < 0.05, ** *p* < 0.01. (**d**,**e**) T cells cultured with ConA and IL-2 were treated with ABT-263 and used for (**d**) TUNEL or (**e**) BODIPY staining. Scale bar: 10 μm. Comparison to wild-type control: NS, statistically not significant; ** *p* < 0.01.

In order to determine whether Alox5 is also important for the induction of caspase-9-independent cell death in vivo, we crossed T/casp9^−/−^ mice with Alox5^−/−^ mice. We did not observe T cell accumulation in T/casp9^−/−^ or Alox5^−/−^ mice compared to wild-type controls (Figure 8a). In contrast, T/casp9^−/−^Alox5^−/−^ mice displayed significant increases in the percentages and total numbers of CD4^+^ and CD8^+^ T cells (Figure 8a), while the numbers of B cells and NK cells were not affected (Figure S2). This indicates that caspase-9- and Alox5-dependent mechanisms can function in parallel to maintain T cell homeostasis in vivo. However, when both pathways are deficient, T cell homeostasis is significantly disrupted. Consistently, wild-type, caspase-9^−/−^ and Alox5^−/−^ T cells treated with etoposide or staurosporine showed similar levels of cell death (Figure 8b). However, caspase-9^−/−^Alox5^−/−^ T cells displayed impaired cell death (Figure 8b). Similarly, ABT-263-induced cell death in caspase-9^−/−^Alox5^−/−^ T cells was significantly reduced compared to that in wild-type, caspase-9^−/−^ and Alox5^−/−^ T cells (Figure 8b). TUNEL staining shows that ABT-263-mediated DNA damage was inhibited in caspase-9^−/−^Alox5^−/−^, but not caspase-9^−/−^ or Alox5^−/−^ T cells (Figure 8c). Treatment with ABT-263 induced increased BODIPY staining in wild-type and caspase-9^−/−^ T cells, but not in Alox5^−/−^ or caspase-9^−/−^Alox5^−/−^ T cells (Figure 8d). While lipid peroxidation was suppressed in both Alox5^−/−^ and caspase-9^−/−^Alox5^−/−^ T cells, only caspase-9^−/−^Alox5^−/−^ T cells showed significant reduction in nuclear DNA damage by TUNEL staining (Figure 8c). In caspase-dependent apoptosis, nuclear translocation of DDF40/CAD after cleavage of DDF45/ICAD leads to DNA fragmentation and apoptosis [7,8,9]. Our data suggest that Alox5 induces lipid peroxidation to promote nuclear damage in a caspase-9-indepenent manner.

### 3.8. Defects in Nuclear Translocation of TIA-1 and EndoG in the Absence of Caspase-9 and Alox5

Caspase-9-independent cell death involved nuclear DNA damages as indicated by TUNEL staining (Figure 1c). Several DNA repair genes, including RAD51 recombinase (RAD51) and Polynucleotide Kinase 3′-Phosphatase (PNKP) [71,72], were identified as candidate cell survival genes against caspase-9-independent cell death (Table S2). We found that silencing of RAD51 or PNKP increased cell death in JMR cells treated with ABT-263 (Figure S4a). Silencing of Apoptosis Inhibitor 5 (API5), an anti-apoptotic gene that may suppress DNA damage [73,74], also increased cell death in JMR cells treated with ABT-263 (Figure S4a). Moreover, silencing of these genes promoted ABT-263-induced DNA damage in JMR cells as shown by TUNEL staining (Figure S4b). These results indicate that the induction of DNA damage is important for the execution of caspase-9-independent cell death.

The protective functions of DNA repair enzymes suggest that DNA damage is important for the execution of caspase-9-independent cell death. In caspase-dependent apoptosis, nuclear translocation of DDF40/CAD after cleavage of DDF45/ICAD leads to DNA fragmentation and apoptosis [7,8,9]. Lipid peroxidation has been shown to promote nuclear translocation of AIF to induce cell death [54]. Endonuclease G (EndoG) and Apoptosis Inducing Factor (AIF) have been shown to enter the nucleus to induce caspase-independent cell death [12,13]. EndoG was among the positive hits of cell death genes in our screening (Table S1). Silencing of EndoG showed an inhibitory effect on caspase-9-independent cell death (Figure S5). In contrast, silencing of AIF did not suppress caspase-9-independent cell death in JMR cells (Figure S5). This could be due to the redundancy of AIF with other effector molecules as well as its homologs [75]. T-cell intracellular antigen-1 (TIA-1) was also a positive hit for cell death promoting genes in our screening (Table S1). TIA-1 is originally identified as a granule-associated RNA-binding protein in cytotoxic T cells that can induce DNA fragmentation in target cells [76]. It is widely expressed in different cell types of lymphoid and non-lymphoid tissues and has been shown to regulate mitochondrial dynamics, apoptosis, autophagy and cell proliferation [77,78]. Consistently, TIA-1 is down-regulated in a variety of human tumors, and its knockdown promotes tumor growth and invasion in mice [79]. We found that silencing of TIA-1 inhibited the killing of JMR cells by ABT-263 (Figure S5), indicating that TIA-1 is an effector molecule to induce caspase-9-independent cell death.

**Figure 9:**
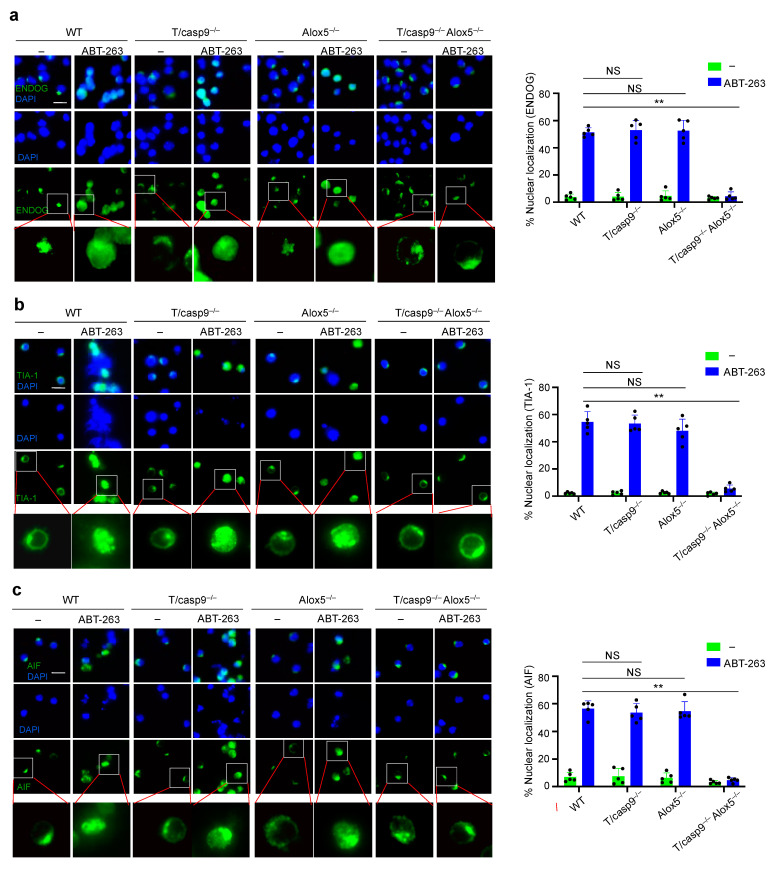
Regulation of nuclear translocation of nucleases and cytolytic molecules by Alox5. T cells cultured as in Figure 8b were treated with ABT-263 for 12 h. The cells were used for immunocytochemistry staining of (**a**) EndoG, (**b**) TIA-1 or (**c**) AIF. The nuclei were counterstained with DAPI. Scale bar: 10 μm. Comparison to wild-type control: NS, statistically not significant; ** *p* < 0.01.

We next examined whether double knockouts of caspase-9 and Alox5 affect nuclear import of cytolytic molecules during cell death. We observed nuclear translocation of EndoG after ABT-263 treatment in T cells from wild-type, T/caspase-9^−/−^ or Alox5^−/−^ mice (Figure 8a). In contrast, T cells from T/caspase-9^−/−^Alox5^−/−^ mice did not display EndoG nuclear translocation after ABT-263 treatment (Figure 9a). Endogenous TIA-1 in primary mouse T cells showed distinct perinuclear localization (Figure 9b). After treatment with ABT-263, nuclear translocation of TIA-1 was observed in wild-type, caspase-9^−/−^ and Alox5^−/−^ T cells, but not in caspase-9^−/−^Alox5^−/−^ T cells (Figure 9b). Nuclear translocation of AIF was also decreased in caspase-9^−/−^Alox5^−/−^ T cells (Figure 9c). Together, these results suggest that Alox5 promotes nuclear entry of cell death-inducing molecules, such as EndoG and TIA-1, to cause DNA damage when caspase-9 is absent.

## 4. Discussion

Through a genome-wide siRNA screening using a caspase-9-deficient Jurkat T cell line, we identified an Erk1-Alox5-mediated cell death pathway in parallel to caspase-9 signaling cascade. Several mitochondrial proteins, including DHODH, HADHA and PDP1, were found to promote ROS production after treatment with a Bcl-2/Bcl-xL inhibitor, ABT-263. Mitochondrial disruption led to ERK1-dependent phosphorylation of Alox5 at Serine 271 and promoted translocation of Alox5 to nuclear membranes. An Alox5 mutant with Serine 271 substitution dominantly interfered with caspase-9-independent cell death. Activation of Alox5 was critical for inducing nuclear translocation of nucleases and cytolytic molecules and the induction of DNA damage. While knockout of caspase-9 in T cells did not cause T cell expansion in mice, double knockouts of caspase-9 and Alox5 in T cells led to severe defects in cell death with significant T cell accumulation. This study suggests that mitochondrial disruption induces the activation of Alox5-dependent membrane lipid peroxidation and translocation of nucleases to the nucleus, resulting in caspase-9-independent cell death. Although the features of caspase-9-independent cell death were grossly similar to the classic caspase-dependent apoptosis with nuclear translocation of DNases and DNA damages, it employed distinct cell death machinery involving oxidative stress, MAPK activation and lipid peroxidation to promote nuclear entry of cell death molecules.

Our genome-wide screening suggests that several sets of genes with opposite functions in regulating oxidative stress and DNA fragmentation also counteract each other in mediating caspase-9-independent cell death. Notably, genes promoting ROS production and oxidative stress, including PDP1, DHODH, HADHA, Alox5 and Alox5AP, were involved in enhancing caspase-9-independent cell death. In contrast, genes with anti-oxidative functions, including MGST1, SESN2 and OXR1, showed significant protection of cell survival. In the downstream, genes involved in DNA fragmentation, such as EndoG and TIA-1, promoted cell death, while DNA repair enzymes, RAD51 and PNKP, inhibited cell death. These data support an important role for oxidative stress and nuclear DNA fragmentation in the induction and execution of caspase-9-independent cell death.

Activation of Erk1 leads to its translocation to the nucleus where it phosphorylates transcription factors and cell cycle regulators to promote cell survival and proliferation [80]. A death-promoting role for Erk independent of caspases has been reported [29,81,82]. Erk1/2 may promote EndoG-mediated cell death independent of caspases [83]. Interestingly, Erk1/2 activation has been associated with oxidative stress injury to neuronal cells in humans and mice [84]. However, the precise molecular mechanisms orchestrated by Erk to promote caspase-independent cell death are not entirely clear. Our data identified a novel mechanism for Erk1 in promoting cell death through the phosphorylation of Alox5. Phosphorylation of Alox5 plays an important in the regulation of its biological activities [63]. We found that mutation of the Ser271 residue in Alox5 impaired nuclear membrane localization of Alox5. This Alox5 mutant also dominantly interfered with caspase-9-independent cell death in JMR cells, suggesting that phosphorylation of Alox5 at Ser271 is critical for the cell death functions of Alox5. After ABT-263 treatment, we detected Erk activation and Alox5 phosphorylation within the first two hours and nuclear membrane translocation within eight hours. This suggests that the Erk1-Alox5 signaling cascade takes place rapidly to mediate caspase-independent cell death. It has been shown that the Serine 271 of Alox5 resides within a nuclear localization signal [65]. The abolishment of nuclear membrane localization by mutating Serine 271 or Erk1 silencing suggests that phosphorylation of this residue is critical for Alox5 translocation during caspase-9-independent cell death.

ROS production and lipid peroxidation have been implicated in apoptosis, autophagic cell death and ferroptosis [85]. Alox5-dependent lipid peroxidation may be important for the execution of cell death triggered by different pathways, through promoting nuclear membrane permeability to facilitate the nuclear translocation of cell death proteins. In intrinsic apoptosis, caspase-9 induces the activation of effector caspases, which in turn cleaves DDF45/ICAD and releases DDF/CAD into the nucleus to cleave DNA [7,9]. Our data suggest that Alox5-mediated lipid peroxidation is important for the nuclear entry of cell death-inducing molecules, EndoG and TIA-1. It has been shown that nuclear entrance of EndoG can lead to cleavage of chromatin DNA independent of caspases [13]. TIA-1 is an RNA-binding protein that regulates pre-mRNA splicing and selective translational silencing [76,86,87,88]. TIA-1 purified from cell lysates or as a recombinant protein can directly induce DNA fragmentation when added to digitonin-permeabilized thymocytes [76], suggesting that it can promote DNA cleavage. TIA-1 can indeed bind to AT-rich DNA in vivo [89]. It has also been shown to be associated with endonuclease PRM1 in stress granules [90]. Whether TIA-1 might promote other nucleases in causing DNA damage remains to be investigated. Knockdown of TIA-1 increased tumor growth and invasion in mice, while TIA-1 expression is down-regulated in a variety of human tumor tissues [79]. Whether the potential tumor suppressor function of TIA-1 is associated with its cell death functions remains to be determined.

A lack of T cell accumulation in T/caspase-9^−/−^ or Alox5^−/−^ mice suggests that caspase-9- and Alox5-dependent cell death mechanisms function in parallel of each other. T cell accumulation in T/caspase-9^−/−^Alox5^−/−^ mice indicates that Alox5-dependent cell death is indeed critical for carrying out intrinsic cell death when caspase-9-dependent pathway is absent. Caspase-9-independent intrinsic cell death is mediated by distinct signaling molecules without the involvement of caspase-9-dependent caspase cascade. Although the early signaling events are different from caspase-dependent apoptosis, the late stages, including nuclear translocation of DNase and DNA damages, are similar. This suggests that cells undergoing such caspase-9-independent intrinsic cell death are efficiently cleared by phagocytosis without causing inflammation.

The Erk1-Alox5-dependent peroxidation of nuclear membrane is a potentially important signaling pathway for the execution of cell death shared by apoptosis and non-apoptotic forms of cell death. Its relationship to apoptosis, necrosis and ferroptosis remains to be determined. This caspase-9-independent cell death mechanism may play an important role in the regulation of diverse biological and pathological processes [85]. Erk-dependent induction of caspase-independent cell death has been observed in neuronal cells [29]. Inhibition of Alox5 translocation has been shown to protect against cerebral ischemia/reperfusion injury [91]. Whether caspase-9-independent mechanisms play a prominent role in neuronal cell death and neurodegeneration will be interesting to investigate. In addition, the induction of caspase-independent cell death is a common feature to many chemotherapeutic drugs [30,31,32,33,34,35,36,37]. Characterization of genes involved in the caspase 9-independent cell death pathways will likely provide more specific and effective targets for cancer therapy.

## Data Availability

All data generated in this study are included in the Figures, Tables and Supplemental Figures and Tables, and are available from corresponding authors.

## Supplementary Materials

The following supporting information can be downloaded at: https://www.mdpi.com/article/10.3390/cells11193053/s1, Figure S1: Transfection of JMR cells with siRNA; Figure S2: Induction of cell death in T cells from T/casp9^−/−^ mice; Figure S3: Lack of T cell accumulation in T/casp9^−/−^ mice; Figure S4: Silencing of DNA damage repair genes promotes cell death in JMR cells; Figure S5: Targeting cell death molecules in JMR cells; Table S1: Candidate cell death genes identified in siRNA library screening; Table S2. Candidate anti-cell death genes identified in siRNA library screening; Table S3: Sequences of siRNAs used in this study.
