# Supplementary material for: Mitochondrion-Mediated Cell Death through Erk1-Alox5 Independent of Caspase-9 Signaling
Source: Cells. 2022 Sep 29;11(19):3053. doi: 10.3390/cells11193053 (PMC9564198; doi:10.3390/cells11193053)
Supplement: Supplementary file 1 [file cells-11-03053-s001.zip › cells-1891236-supplementary.pdf]

Supplementary Figures and Tables

Mitochondrion-mediated Cell Death through Erk1-Alox5 Independent of Caspase-9 Signaling

Min Chen[1,*], Lei Wang[1], Huanhuan Sun[1], Min Li[2], Marietta M. Budai[2] and Jin Wang[2,3*]

[1]Department of Pathology and Immunology, Baylor College of Medicine, Houston, TX 77030, USA

[2]Immunobiology and Transplant Science Center, Houston Methodist Research Institute, Houston, TX 77030, USA

[3]Department of Surgery, Weill Cornell Medical College, Cornell University, New York, NY 10065, USA

*Correspondence: minc@bcm.edu (M.C.); jinwang@houstonmethodist.org (J.W.)

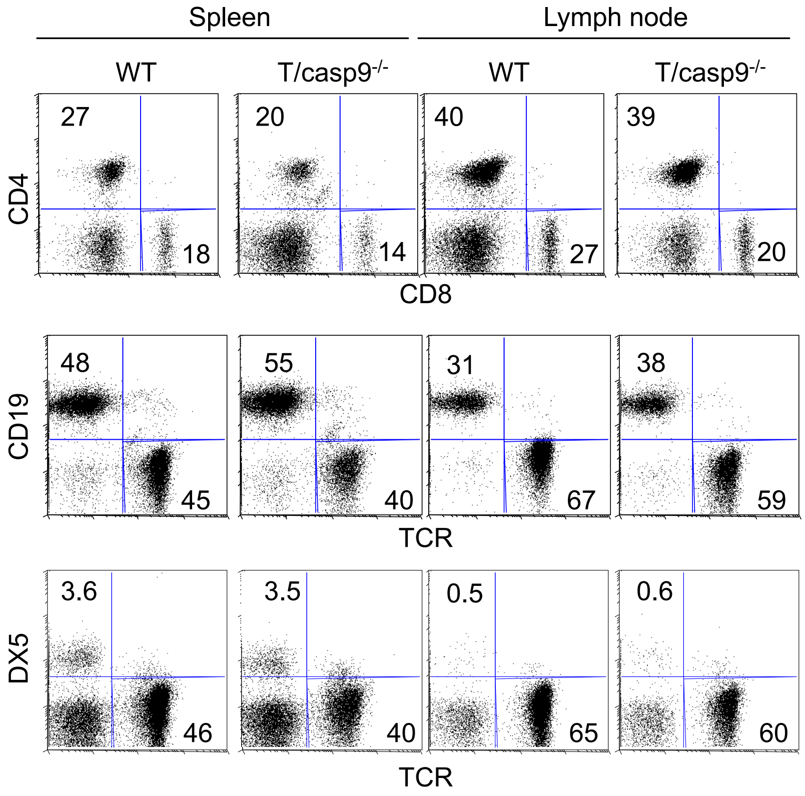

Figure S1. Lack of T cell accumulation in T/casp9$^{-/-}$ mice. Caspase-9^{flox} mice were crossed with lck-cre mice to generate T/caspase-9$^{-/-}$ (T/casp9$^{-/-}$) mice. Flow cytometry of the cells from the spleen and lymph nodes of 3-month-old T/casp9$^{-/-}$mice and wild type (WT) controls was performed to analyze the following cell types: TCR^{+} T cells, CD19^{+} B cells, CD4^{+} or CD8^{+} T cells and DX5^{+} NK cells.

\

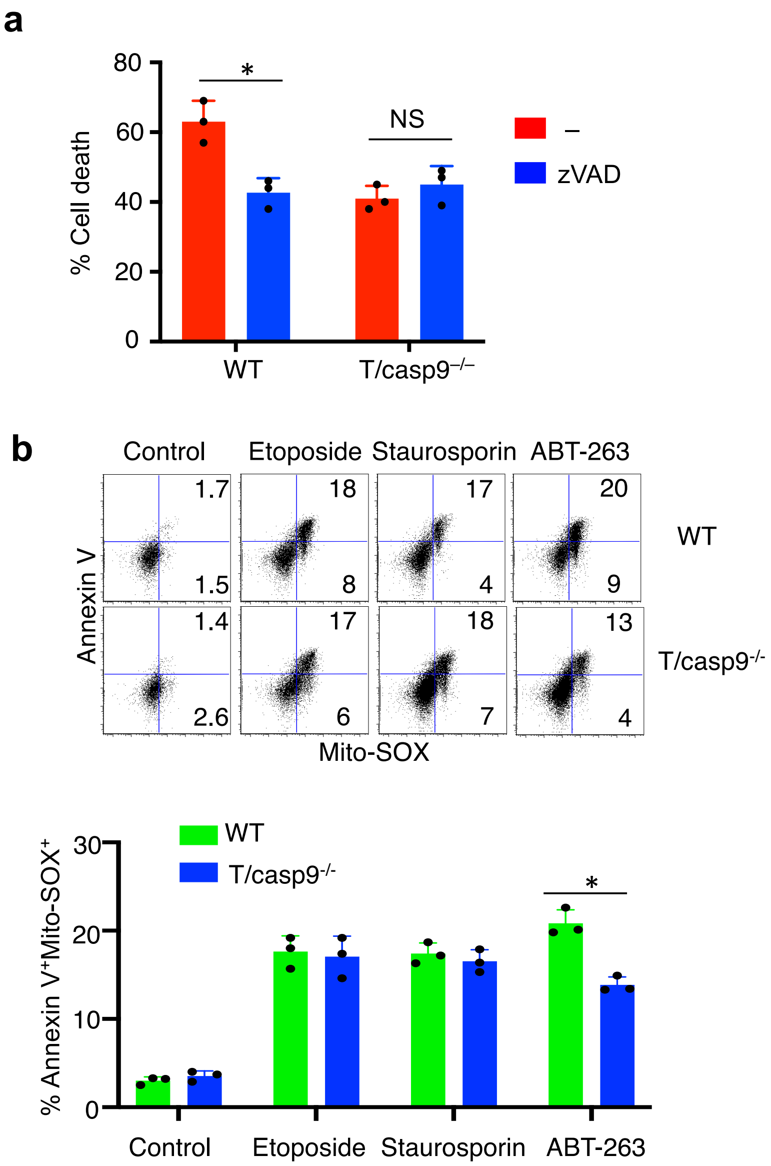

Figure S2. Induction of cell death in T cells from T/casp9$^{-/-}$ mice. (a) T cells from wild type and T/casp9$^{-/-}$ mice were cultured with 1 μM ABT-263 with or without zVAD for 24 h. *P<0.05; NS, statistically not significant. (b) Staining with Annexin V and Mito-SOX in wild type (WT) and casp9$^{-/-}$ T cells. Wild type and casp9$^{-/-}$ T cells were treated with etoposide (1 μM), staurosporin (10 nM) or ABT-263 (1 μM) for 6 h. The cells were stained with mito-SOX and APC Annexin V, and analyzed by flow cytometry. *P<0.05.

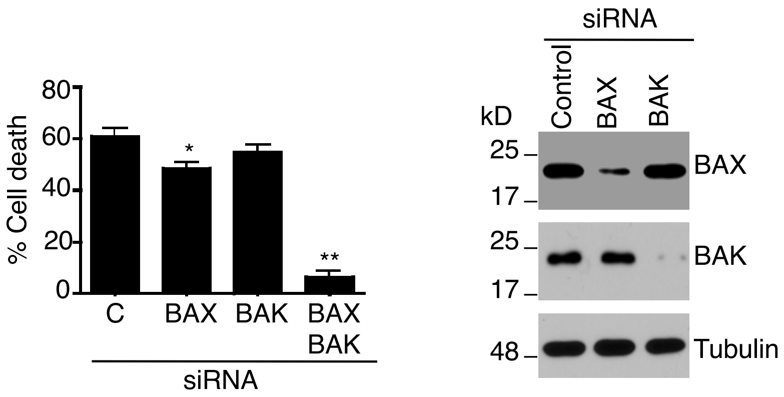

Figure S3. Transfection of JMR cells with siRNA. JMR cells were transfected with Alexa Fluro 488-conjugated siRNA (Qiagen) using RNAiMax (Life Technologies) and analyzed by flow cytometry. Dashed line: untransfected control. JMR cells were also transfected with siRNA targeting BAX and BAK. The cells were used to determine ABT-263-induced cell death or Western blot analysis. Data are presented as mean ± SD. Comparison to control: *P<0.05, **P<0.01.

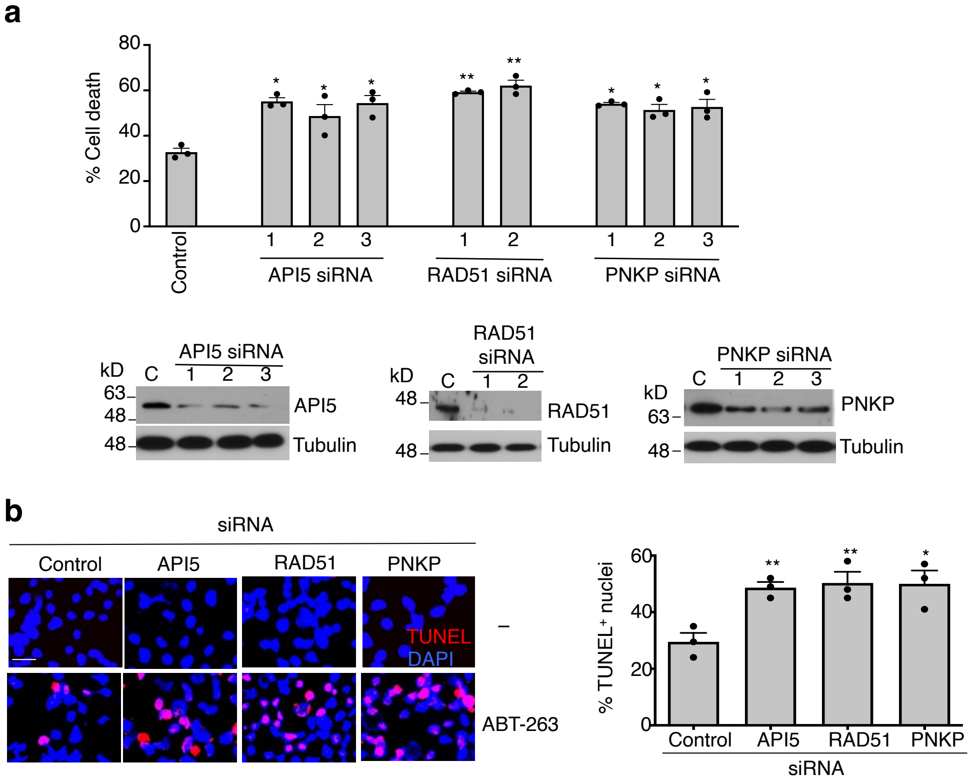

Figure S4. DNA damage repair genes in the inhibition of cell death in JMR cells. (a) JMR cells transfected with individual siRNA targeting different genes or non-targeting control (c) were treated with ABT-263, followed by quantitation of cell death (upper panels). Data are presented as mean ± SD. *P<0.05, **P<0.01. Western blot was also performed for the transfected cells (lower panels). (b) Cells treated as in (a) were also used for TUNEL staining.

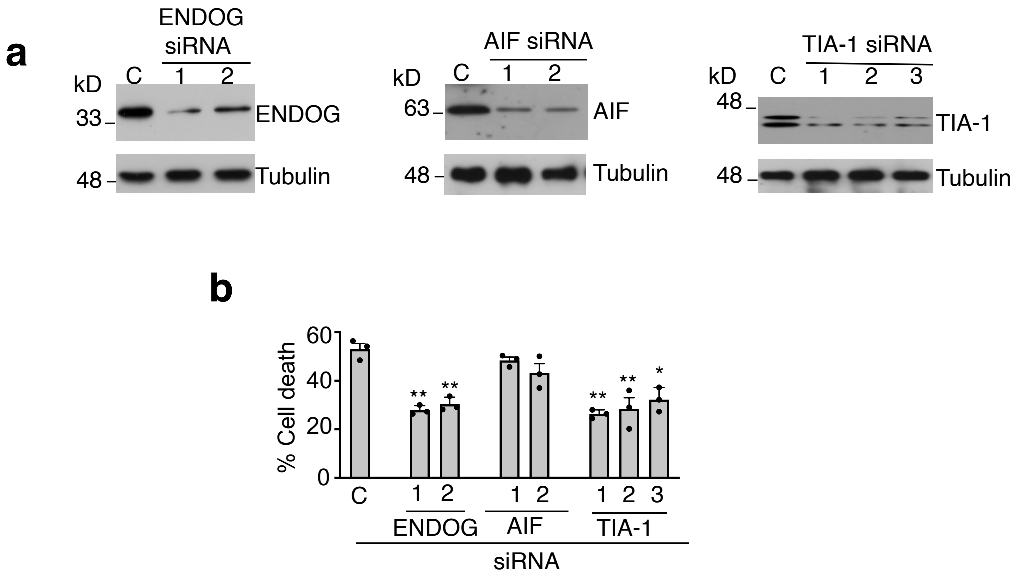

Figure S5. Targeting cell death molecules in JMR cells. (a) JMR cells transfected with individual siRNA targeting TIA-1, EndoG or AIF and analyses by Western blot. (b) The cells were used to determine ABT-263-induced cell death. Data are presented as mean ± SD. Comparison to control: *P<0.05, **P<0.01.

Table S1. Candidate cell death genes identified in siRNA library screening.

Gene symbol	Clasification
KIAA0261	Cell Cycle regulation
SMC1L2	Cell Cycle regulation
PRM3	Cell Cycle regulation
ERAF	Chaperone
HSPA8	Chaperone
ERBP	Chromatin remodeling
TIA1	Cytolytic/nuclease
WBSCR22	DNA Methyltransferase
ENDOG	Endonuclease
MLL2	Histone methyltransferase
MAPK3	Kinase
MAP3K3	Kinase
MAPK6	Kinase
MAP4K3	Kinase
PAK1	Kinase
TEK	Kinase
ROS1	Kinase
CAMKIINALPHA	Kinase regulator
RRAD	Kinase regulator
HUNK	Kinase regulator
GRHPR	Metabolism
UGCG	Metabolism
PDE5A	Metabolism
MGC20781	Metabolism
MGC23940	Metabolism
DHODH	Mitochondrial
HADHA	Mitochondrial
PDP1	Mitochondrial
LDHA	Mitochondrial
BAX	Mitochondrial/Pro-apoptotic
ULBP3	Other/Calcium mobilization
HSGT1	Other/p53 regulator
ANGPT1	Other/TEK/TIE2 receptor activation
FLJ38822	Oxidative killing
ALOX5	Oxidative stress
ALOX5AP	Oxidative stress
NP	Peptidase
THY28	Pro-apoptotic
DDX47	Pro-apoptotic
IGFBP3	Pro-apoptotic
DKFZP547E052	Protease
GULP1	Protein trafficking
LMAN2L	Protein trafficking
AP1S3	Protein trafficking
FLJ30990	Protein trafficking
COPB2	Protein trafficking
RABGGTA	Protein trafficking
RNASE8	Ribonuclease
DDX27	RNA helicase
SCA1	RNA metabolism
EXOSC1	RNA processing
DHX8	RNA splicing
PRPF8	RNA splicing
SFPQ	RNA splicing
PLRG1	RNA splicing
RGS20	Signaling/G protein
RASAL1	Small G protein regulator
RASGRF2	Small G protein regulator
RASGRP1	Small G protein regulator
CBFA2T1	Transcription factor
KLF3	Transcription factor
TEX10	Transcription factor
ZNF42	Transcription factor
SERTAD2	Transcription factor
MGC27466	Transcription factor
TSC2	Tumor suppressor
WDR11	Tumor suppressor
KIAA0953	Unknown
THAP6	Unknown
DCBLD1	Unknown

Table S2. Candidate anti-cell death genes identified in siRNA library screening.

Gene symbols	Classification
API5	Anti-apoptotic
BCL2	Anti-apoptotic
BCL2L1	Anti-apoptotic
FMNL1	Anti-apoptotic/proliferative
TIC	Anti-apoptotic
ETUDE	Anti-oxidative
MGST1	Anti-oxidative
OXR1	anti-oxidative
SESN2	anti-oxidative
C10ORF9	cell cycle
PRCC	cell cycle
KIAA0542	Cell cycle/dynamic structure of centrosome
KIAA0056	Cell cycle/mitotic chromosome assembly
384D8-2	Cell cycle/mitotic chromosome assembly
NELL2	Cell growth and differentiation/neuron
DRD3	Cell proliferation
FGF10	Cell proliferation and celldifferentiation
RAD51	DNA damage repair
PNKP	DNA damage repair
MORF4L2	Histone acetyltransferase
UTX	Histone demethylase
FBXL11	Histone demethylase
USP21	Histone H2A/Deubiquitinates
MGC10731	Membrane trafficking
ADHFE1	Metabiolism
PRPSAP1	Metabiolism
PYGL	Metabiolism
GBA2	Metabiolism
B4GALT2	Metabolism/glycosylation
CPT1B	Mitochondrial
NDUFB5	Mitochondrial
NELL2	Other/Neural cell growth and differentiation
NCDN	Other/negative regulator of CaAMKII
PPIF	Protein folding
SIRT2	Protein modification/Deacetylase
CGI-100	Protein trafficking
CGI-100	Protein trafficking
SLC9A3R2	Scaffold protein
ZFP161	Transcription factor
MTF1	Transcription factor
PLAGL2	Transcription factor
NFIC	Transcription factor
PITX3	Transcription factor
ID1	Transcription factor
LOC390284	Unknown
LOC400050	Unknown
LOC402475	Unknown
THSD6	Unknown
FLJ22160	Unknown
VPS24	Unknown
DNM1	Vesicular trafficking
STX16	Vesicular transpor
STX10	Vesicular transport

Table S3. Sequences of siRNAs used in this study.

Gene symbol	siRNA#	siRNA Sequence
DHODH	1	GGUAUGGAUUUAACAGUCATT
DHODH	2	AGGAAACCCUAGACCCAGATT
DHODH	3	CGGGAUUUAUCAACUCAAATT
HADHA	1	CCGUCCUUAUCUCAUCAAATT
HADHA	2	GGACAAUAGAAUACCUAGATT
PDP1	1	CAACGAGUUUGGGACUGUUTT
PDP1	2	CAGUCACGCUGUCUAAUGATT
PDP1	3	CGCAAGUUGGUGAUCCUAATT
MGST1	1	CAUUUCUUGGAAUUGGCCUCCUGUA
MGST1	2	CCAAGAAGUAUCUUCGAACAGAUGA
MGST1	3	GAUGCUUAUGAGUACUGCAACUGCA
SESN2	1	GCUACUCGCUGAUCCAGCGGCUUUA
SESN2	2	UCCGCAGGGCCAUCUGGAACUAUAU
SESN2	3	CCCAGACAUGCUGUGCUUUGUGGAA
OXR1	1	CCAUGGAAGAAGCCAUUCUUGUAAA
OXR1	2	UGGCUAUCCAUGGACUCUUGUUUAU
OXR1	3	GGAAGAUCAGAUUGCAGAUAACUUU
SOD1	1	GGAAGUCGUUUGGCUUGUGGUGUAA
SOD1	2	CCAUGUUCAUGAGUUUGGAGAUAAU
SOD1	3	GACUGACUGAAGGCCUGCAUGGAUU
Alox5	1	GCACAUGUUCCAGUCUUCUUGGAAU
Alox5	2	CCGCUCCCAUCUGCUUGCUGUAUAA
Alox5	3	GAGUACCUGACCGUGGUGAUCUUCA
Alox5AP	1	CCUGCUGCGUUUGCUGGACUGAUGU
Alox5AP	2	UCCGUUGCUGGCAUAUUCAACUAUU
Alox5AP	3	ACACUUGCCUUUGAGCGGGUCUACA
Erk1	1	GGAUCAGCUCAACCACAUUTT
Erk1	2	GGACCGGAUGUUAACCUUUTT
API5	1	GCAGCUCAAUUUAUUCCGATT
API5	2	CCACAAGGUUUGUGACAUATT
API5	3	CGAGCAAUUAAAUUCCUUUTT
NEIL1	1	GGCUCGCCCUAUGUUUCGUGGACAU
NEIL1	2	GGCUGCGUGGAGAAGUCCUCUGUCA
NEIL1	3	GCCAGCCAGUUUGUGAAUGAGGCCU
RAD51	1	CCACCAGACCCAGCUCCUUUAUCAA
RAD51	2	GCGACUCGCUGAUGAGUUUGGUGUA
PNKP	1	GCCACAACAACCGGUUUCGAGAGAU
PNKP	2	UGAGACAGCCCUGAAGCAAGGGAAA
PNKP	3	GGAAGUCCACCUUUCUCAAGAAGCA
TIA-1	1	GCGUCAGACUUUUUCACCATT
TIA-1	2	GAUAUUCAUUUGUUCGGUUTT
TIA-1	3	CGCUCCAAAGAGUACAUAUTT
ENDOG	1	GGAACAACCUGGAGAAAUATT
ENDOG	2	GCAGCUACCAAAACGUCUATT
AIFM1	1	GGAACAUCUUUAACCGAAUTT
AIFM1	2	GCAUGCUUCUACGAUAUAATT
